# 927. Clinical Characteristics and Outcomes of Norovirus Infection in Patients with Hematologic Malignancies: A Retrospective, Single Center Study

**DOI:** 10.1093/ofid/ofab466.1122

**Published:** 2021-12-04

**Authors:** Taylor Brooks, Thomas A Crilley, Gregory B Russell, Bhavita Gaglani, Niyati Jakharia

**Affiliations:** 1 Wake Forest Baptist Medical Center, Winston-Salem, North Carolina; 2 University of Maryland, Laurel, Maryland

## Abstract

**Background:**

Norovirus (NV) gastroenteritis has been identified as a cause of significant morbidity among hematopoietic stem cell transplant (HSCT) recipients, often with associated complications. Current guidelines recommend symptomatic relief with antimotility agents, rehydration, and reduction in immune suppression. Nitazoxanide (NTZ) is an anti-parasitic agent but some literature suggests a benefit of nitazoxanide therapy for NV.

**Methods:**

We conducted a single center, retrospective chart review study and evaluated adult patients (age >18 years) who had NV infection and either: 1) underwent stem cell transplantation; or 2) received myeloablative chemotherapy within 4 weeks of NV diagnosis by positive test on gastrointestinal pathogen panel during the time period from January 2015 through March 2020.

**Results:**

26 patients were reviewed. 14 patients (54%) had a history of HCST prior to infection. Three patients (12%) received both myeloablative chemotherapy and HSCT within four weeks of NV infection. Six patients (46%) had autologous, six (46%) had matched unrelated donor, and one (8%) had haploidentical allogeneic transplants. Nine (69%), three (23%), and one (8%) underwent myeloablative, reduced intensity and non-myeloablative conditioning, respectively. Median duration of diarrhea was 4.5 days (IQR = 2.25-7 days). Three (12%) patients received NTZ or intravenous immune globulin. The 6 month mortality was 42% (11/26), however, none of the deaths were directly attributable to NV infection.

**Conclusion:**

NV infection led to severe diarrheal disease in our cohort. Overall mortality was high, and a trend toward increased mortality was seen among patients receiving NV-directed therapy; these patients likely received NV-directed therapy due to the severity of their illness. Clinicians must have a high suspicion for this illness and obtain PCR testing for timely diagnosis and management.

Table 1. Characteristics of patients with hematologic malignancies and norovirus infection

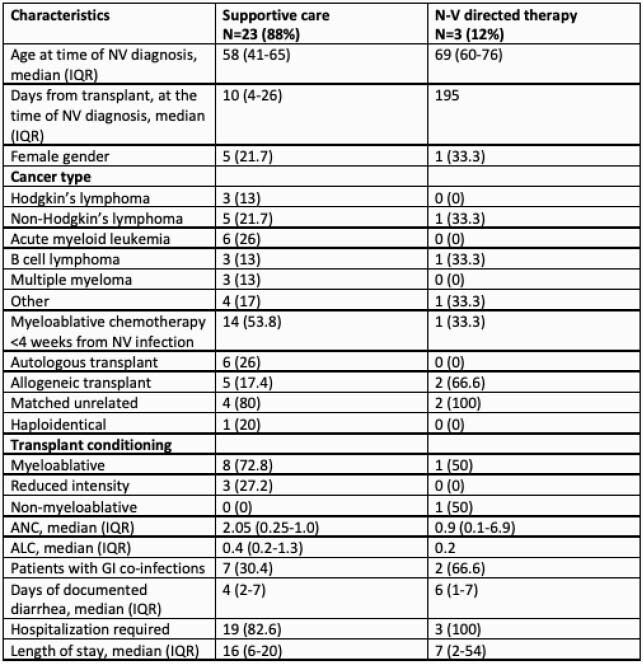

**Disclosures:**

**All Authors**: No reported disclosures

